# A serious game for assessing upper-limb visuomotor adaptation in children with cerebral palsy during reaching tasks in virtual reality

**DOI:** 10.1038/s41598-026-54191-y

**Published:** 2026-05-25

**Authors:** Guido Pasquini, Roberto Maria Scardigno, Domenico Buongiorno, Vladimiro Suglia, Laura Antonucci, Sara Della Bella, Chiara Beni, Nicole Lonoce, Paola Carrozza, Claudio Macchi, Adriano Ferrari, Giovanna Cristella, Vitoantonio Bevilacqua

**Affiliations:** 1 IRCCS Fondazione Don Carlo Gnocchi ETS, Florence, 50143 Italy; 2 Department of Electrical and Information Engineering (DEI), Polytechnic University of Bari, Bari, 70126 Italy; 3 University of Modena and Reggio Emilia, Modena, Italy

**Keywords:** Health care, Neurology, Neuroscience

## Abstract

Cerebral palsy (CP) is the most common non-progressive neurodevelopmental disorder, associated with impairments in motor control, posture, and adaptability. Understanding how patients with CP adapt to different task demands is essential to design effective assessment and rehabilitation tools. The aim of this study is to provide a detailed characterization of the movement patterns observed between a group of patients with CP and a Control Group, with the goal of identifying specific motor control features that may influence adaptability in several mapping conditions of the game. A cohort of 15 individuals with CP and 10 controls performed game-based tasks under varying mapping conditions. Kinematic and performance metrics were extracted and analyzed to quantify differences in motor strategies, variability, and adaptability across groups. Patients with CP showed distinct movement patterns compared to controls, particularly in adaptability metrics. The adaptability of motor control was influenced by the mapping condition, revealing group-specific limitations. This study highlights characteristic motor control features in CP that influence adaptability to task constraints. These findings may inform the development of personalized rehabilitation protocols and adaptive game-based interventions for motor training.

## Introduction

Cerebral palsy (CP) is the most common non-progressive neurodevelopmental disorder, caused by a brain injury that occurs during pregnancy, at birth, or after birth, and results in motor and postural function impairments^1^. The motor disorders include poor coordination, spasticity, seizures, mental deficiency, and disturbances affecting perception, vision, hearing, swallowing, and speaking^2–4^. In recent years, increasing attention has been directed towards the non-motor signs and symptoms of CP, underlining the role of cognitive impairment and perceptive alteration in influencing motor control and recovery possibilities^5^. CP represents the most common motor disability in childhood, with a prevalence rate of nearly 1.2 per 1000 live births^6–8^ and has a significant impact on daily-life motor actions such as gait, manipulation, and reaching^9–11^.

Several therapeutic interventions are used in the management of CP; in particular, the most common techniques are traditional physiotherapy and occupational therapy, both tailored to the patient’s individual needs. Some of the main goals of rehabilitation treatment are given by functional independence and mobility, but communication and social skills are pursued in addition to motor skills. Nevertheless, the repetitive nature of traditional physical exercises often leads to reduced patient engagement, thus jeopardizing the effectiveness of rehabilitation treatment^12,13^. The introduction of virtual reality (VR) and serious games (SGs) in the traditional approach helps to raise the child’s compliance, offering a tailored program through a realistic and adaptable environment^14,15^. In fact, VR allows increasing motivation of CP patients by integrating with gamification strategies in gait training^16,17^, balancing exercises^18,19^, upper-limb motor control^10,20^, and reaching tasks^21–25^. Despite growing interest in VR-based rehabilitation for individuals with CP, most of the existing systems emphasize performance metrics, often overlooking how users adapt their motor behavior in response to altered visual feedback.

Visuomotor adaptation (VMA) paradigms provide a valuable framework for studying sensorimotor plasticity and mechanisms for error corrections^26^. These processes are relevant in populations with impaired motor planning and execution, such as those with CP^27^. Therefore, integrating VMA paradigms into VR-based studies can deepen our understanding of motor behavior changes in response to mismatches between intentional movements and sensory outcomes^24,27^. Hence, VR enables a fully customizable mapping between the physical and virtual worlds through visual perturbations, which can be implemented realistically and easily adapted to the desired protocol^26^.

A large variety of studies have incorporated VMA paradigms within VR-based frameworks for clinical assessment and rehabilitation. Specifically, several VR systems have employed visual perturbations, such as angular deviations between visual feedback and executed motor commands, to evaluate motor functions in individuals with neuromotor disorders, including dystonia^28^, Parkinson’s disease^29^, essential tremor^30^, and multiple sclerosis^31^. Furthermore, other investigations have examined the impact of aging on motor^32^ and cognitive^33^ performances by introducing oscillatory visual distortions (e.g., sinusoidal shifts in the visual field) to probe age-related declines in sensorimotor integration.

Despite these advances, there remains a marked paucity of research applying VMA paradigms to assess motor capabilities in individuals with CP. To the best of our knowledge, only a single study has integrated VMA within a SG-based environment for this population. In this work, Cristella et al. assessed the sense of position in individuals with spastic diplegic CP by introducing visual perturbations that distorted the correspondence between physical and virtual hand trajectories while performing reaching tasks^5^. This evident gap in the literature underscores the need for further investigations regarding the application of visual perturbations in VR-based serious games to elicit and evaluate VMA in individuals with CP.

Building upon this context, this study introduces a novel VR-based serious game designed to evaluate visuomotor adaptation capabilities in individuals with CP during upper-limb reaching tasks. The game, conceptually inspired by the arcade classic “Whack-a-Mole”, requires the control of a virtual hammer with reaching movements to strike targets (“moles”) that appear at varying spatial locations within a VR scenario displayed on a 2D monitor. Participants’ engagement is enhanced through the use of visual perturbations that are specifically designed to challenge visuomotor coordination, and whose impact is quantitatively evaluated using a set of extracted performance features.

The aim of this study is to provide a detailed characterization of the movement patterns observed between a group of patients with cerebral palsy and a Control Group, with the goal of identifying specific motor control features that may influence adaptability in several mapping conditions of the game.

## Materials and methods

This section presents in detail the proposed VR-based framework along with the considered performance metrics, the experiment design, and the conducted statistical analyses.

### The VR-based framework

This subsection outlines the proposed framework, detailing the setup and tracking system, the fully customizable VR-based serious game, and the calibration procedures.

The framework was developed based on a set of technical requirements defined through collaborative meetings between the academic team and the clinical staff at IRCCS Fondazione Don Carlo Gnocchi ETS (Florence, Italy). The clinical team expressed the need for a customizable VR-based SG capable of assessing VMA in children with neuromotor disorders during upper-limb reaching tasks. To guide the design and implementation of the proposed system, the following requirements were established:

- the game must remain simple and intuitive to ensure sustained engagement from pediatric users;
- gameplay should occur within a limited movement space, minimizing compensatory strategies and involving only the upper limb;
- the system should include a calibration phase to account for the different anthropometric characteristics of users’ upper limbs, as these can differ significantly across individuals;
- the participant must control the position of a virtual hammer within a customized virtual environment while performing reaching tasks;
- the system must implement two visuomotor perturbations affecting the mapping between the real and virtual hand positions along the mediolateral (ML) axis, *Gain*, which amplifies the virtual motion, and *Reversal*, which inverts its direction;
- a markerless motion tracking system should be adopted to streamline the setup process and reduce encumbrances.

#### The setup

**Fig. 1:**
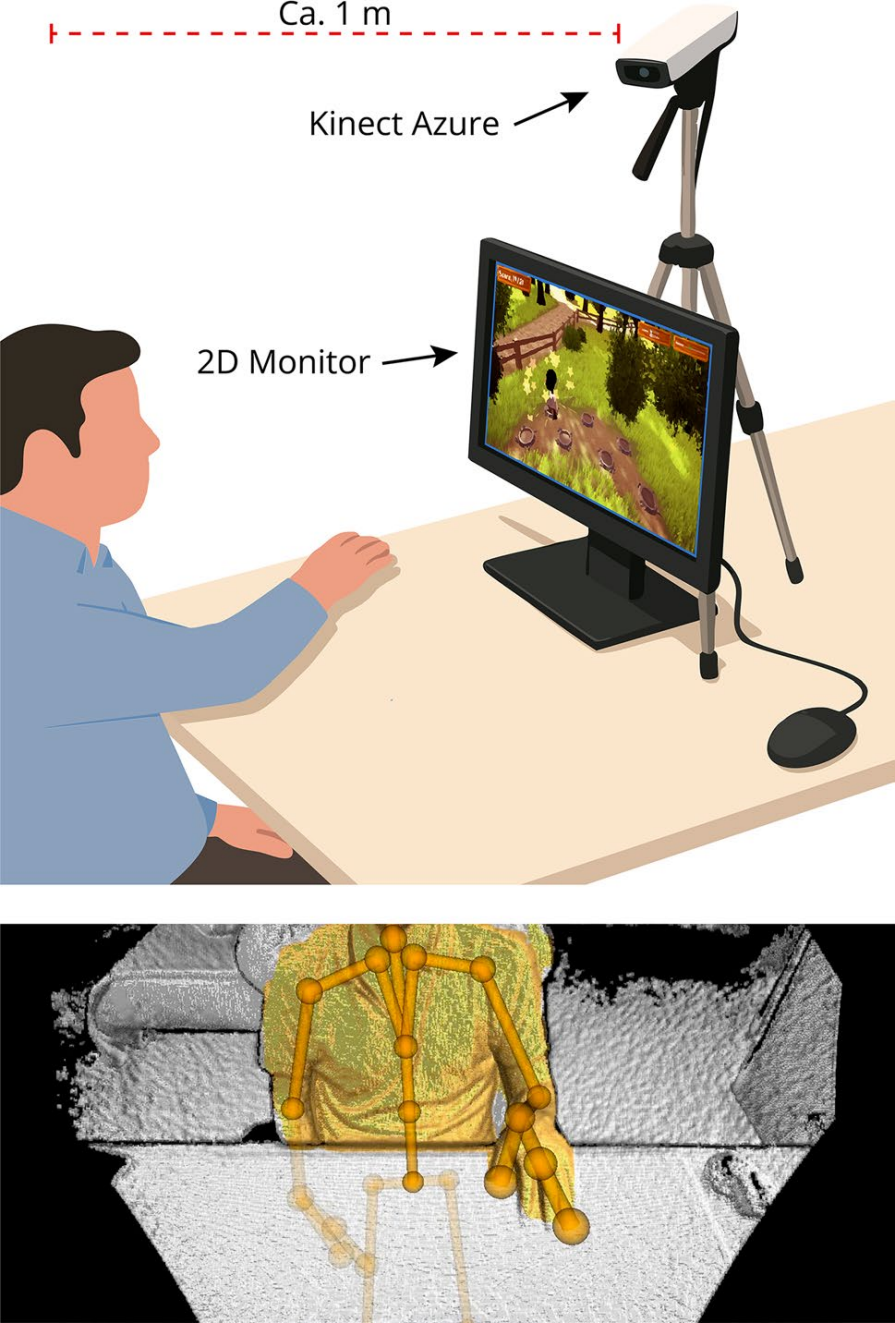
Top. The setup of the proposed framework. Bottom. The skeleton extracted by using the Kinect Azure camera.

As shown in Fig. 1, the participant is seated on a chair in front of a 2D monitor, with the shoulder of the active limb positioned as close as possible to the edge of the desk and aligned with the center of the screen. This positioning allows for a natural reaching posture. Sufficient distance from the monitor is maintained to enable free movement within the virtual play area, without physical obstructions. The same fixed distance from the monitor is maintained across all participants, as it serves only to provide approximate feedback on the relative position between the virtual hand and the target.

#### The proposed serious game

The proposed SG, inspired by the classic arcade game named “Whack-a-Mole”, is designed to reward participants for striking virtual moles using a virtual hammer controlled through movements of the arm. Participant engagement is reinforced through a simple scoring mechanism, defined as follows:

- 1 point is awarded if the mole is successfully struck within a predefined time window;
- 0 points are assigned if the mole is not hit before the timeout expires.

The virtual camera remains fixed on the array of mole holes, and the participant is instructed to maintain a stable seated posture to ensure accurate motion tracking. Interaction with the moles is only enabled after the participant has grasped the virtual hammer, which is initially located at a specific home position. This constraint ensures that each reaching movement consistently begins from a central point and terminates at the position of the selected target mole, with the aim to enable fair intra-subject and inter-subject comparisons.

Each attempt to reach and strike a mole constitutes a single trial. Upon successfully hitting a mole, the hammer position is automatically reset to the home position. A pulsing animation is then applied to the hammer, serving as a visual cue to prompt the participant to grasp it and initiate the next trial.

**Fig. 2:**
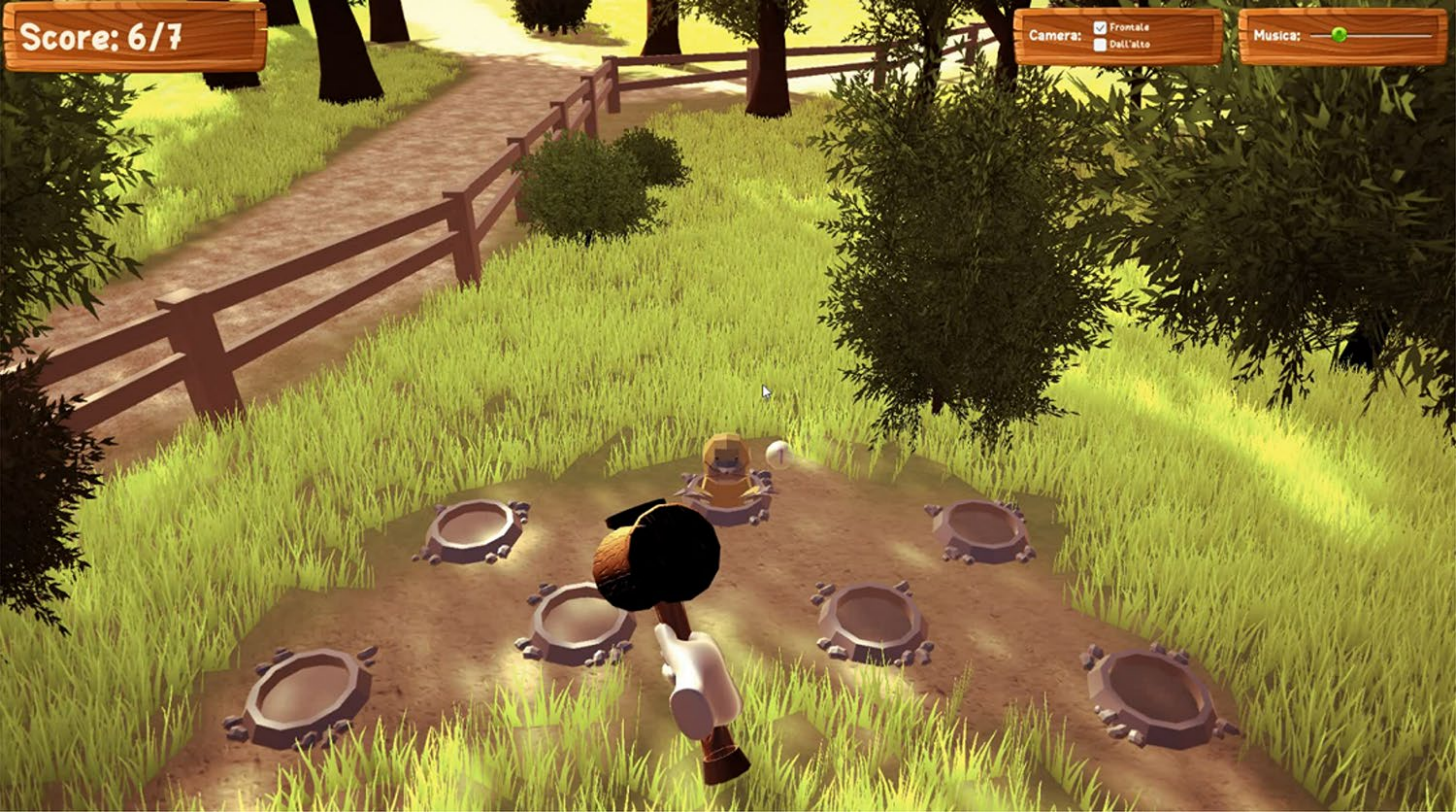
A scene of the serious game.

The virtual targets are placed at fixed locations within the play area, which is designed to approximate a semicircular region reachable by a healthy participant without compensatory movements. Specifically, this region allows for full arm extension and internal/external humeral rotation within the transverse plane, and it is arranged to be as symmetrical as possible with respect to the dominant shoulder. The seven mole positions span the entire area, as shown in Fig. 2, requiring a range of movement representative of typical upper-limb reaching tasks.

**Fig. 3:**
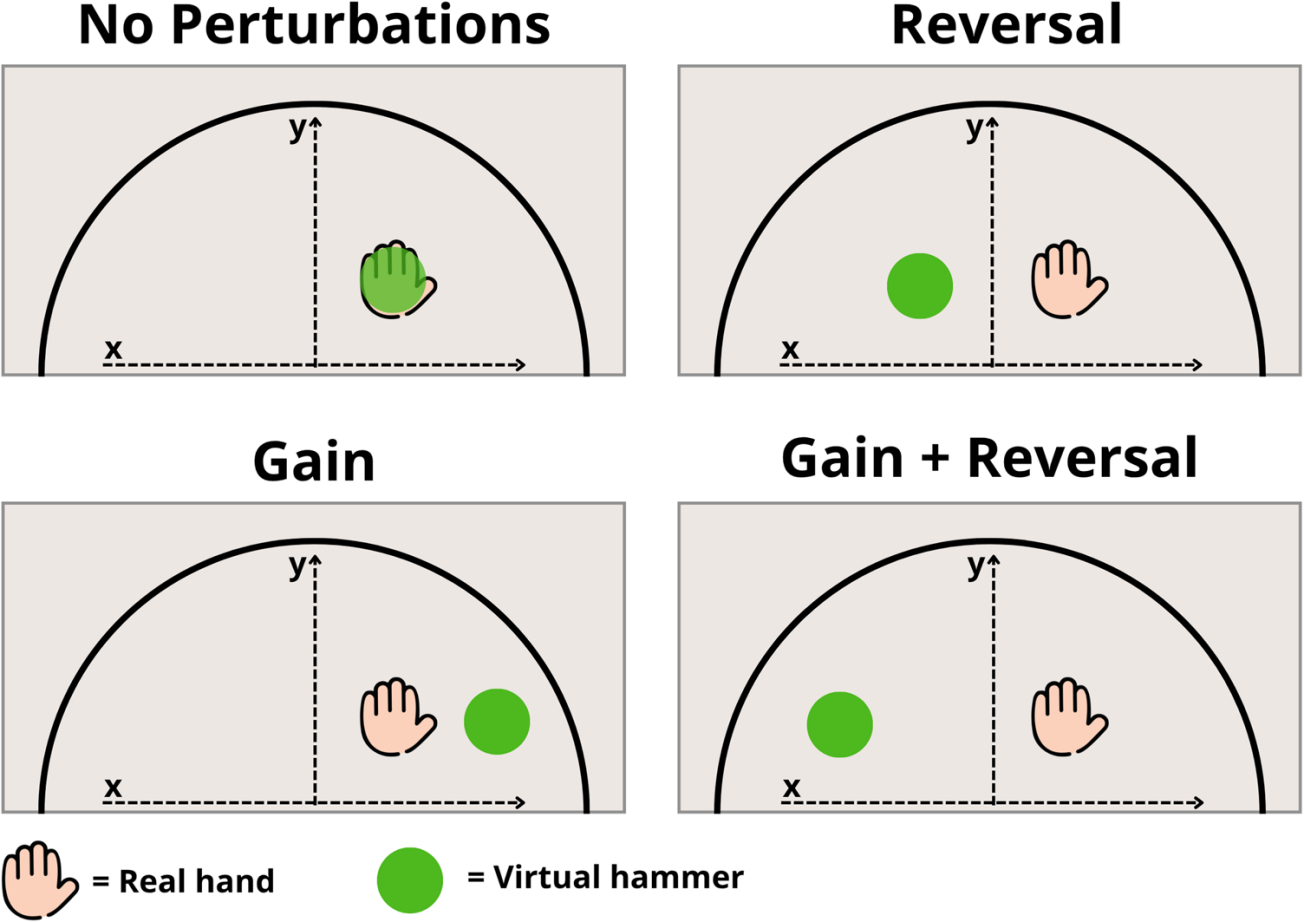
Illustration of the four visuomotor mapping conditions implemented across the experimental blocks. Each panel shows the spatial relationship between the real hand (depicted as a palm icon) and the virtual hammer (green circle) along the ML (x) and AP (y) axes of the real and virtual environment, respectively.

Furthermore, real and virtual displacements are mapped such that the boundaries of the virtual environment correspond to the physical workspace defined by the participant’s range of motion along the mediolateral and anteroposterior (AP) axes. This mapping, which serves as the baseline condition for the game in which no visuomotor perturbations are applied, consists of applying a scaling factor to the real 3D displacement components, as described in the ’Hand Calibration’ section below. The aforementioned visual perturbations, namely, *Gain* and *Reversal*, can be applied individually or in combination within a single trial to modify the mapping between the virtual hand position and the actual position of the participant’s hand. Accordingly, four distinct mapping conditions have been implemented (see Fig. 3):

1. *No Perturbations*: no modification is applied to the mapping; the virtual hand moves concordantly with the physical hand. For example, when the participant moves their hand to the left or right boundary of the physical workspace, the virtual hand reaches the corresponding side of the play area.
2. *Reversal*: the ML displacement is inverted, such that moving the physical hand to the left results in the virtual hand moving to the right, and vice versa.
3. *Gain*: the ML displacement is amplified while preserving directional consistency. Specifically, the virtual hand moves in the same direction as the physical hand, but its displacement is scaled by a predefined gain factor, set to 2.
4. *Reversal+Gain*: the mapping is perturbed through the simultaneous application of *Reversal* and *Gain*, thus reverting and amplifying the virtual hand position at the same time.

The SG includes different soundtracks to enhance player engagement and prevent boredom. Remarkably, a user-friendly interface is embedded to allow clinicians to build a customized experimental protocol, i.e., the types of visual perturbation addressing VMA.

This SG offers the advantage of scalable development, with the incorporation of progressively more challenging levels of difficulty. It was developed using the Unity3D framework (release 2022.1.8f1, Unity Technologies, San Francisco, CA, USA). The Sample Unity Body Tracking Application (GitHub Commit Number: d87e80a, Microsoft, Redmond, WA, USA)^34^, is exploited as base to link the motion of human joints, tracked with the Kinect Azure SDK (version 1.4.1, Microsoft, Redmond, WA, USA)^34^, to the corresponding virtual hand motion.

#### The tracking system

The subject controls the position of the virtual hand through a real-time body-tracking system. Specifically, the visible part of the participant’s body is captured using a 3D Azure Kinect sensor operating at a frame rate of 50 Hz^34^. The sensor is mounted on a tripod positioned approximately 1 m in front of the participant, with its height adjusted to about 20 cm above the subject’s head. Additionally, the device is tilted downward to ensure that the entire play area is covered while minimizing occlusions from surrounding objects. An overview of the proposed setup is shown in Fig.  1.

#### Initial calibration procedure

An initial calibration procedure, composed of two sub-phases, i.e., the environmental setup and the hand calibration, has been introduced to define the pose (position and orientation) of the virtual space with respect to the real space, and to adapt the dimension of the real play space to the player’s upper limb anthropometric characteristics.

**Fig. 4:**
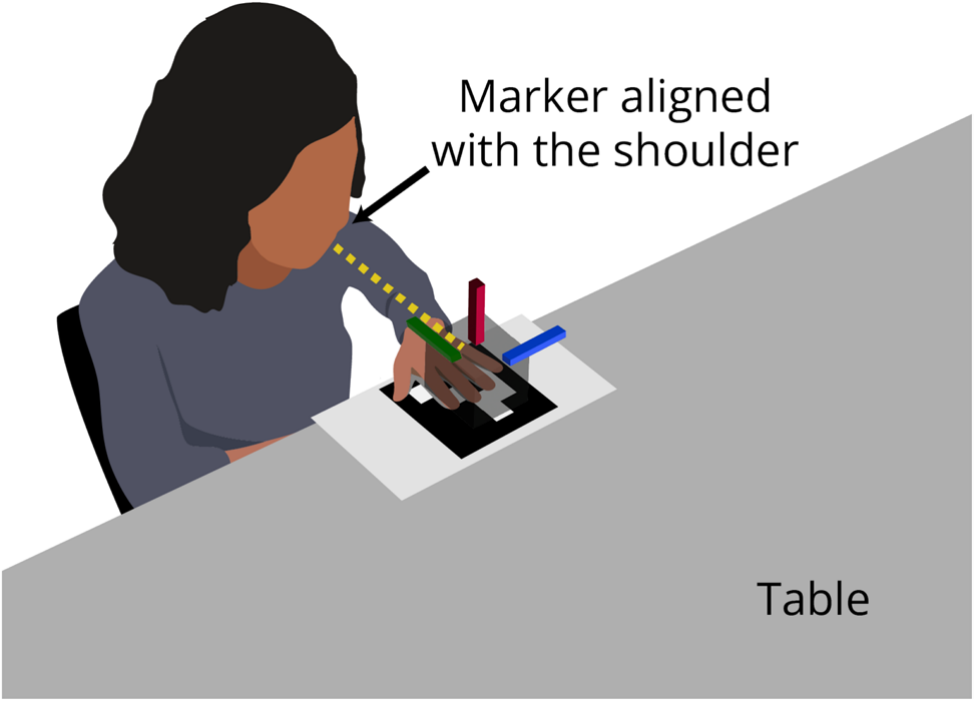
Calibration procedure using an ArUco marker to align the virtual and physical environments. During this phase, the relative pose between the Azure Kinect camera and the participant is estimated using a printed ArUco marker placed on the table near the dominant shoulder.

**Environment setup.** This phase of the calibration procedure is necessary to correctly define the position and orientation of the virtual game space within the real 3D environment. In other words, it allows the virtual moles to be placed at the correct predefined positions in the real space. A virtual 3D reference system is placed on the table as close as possible to the participant’s right or left shoulder, depending on the hand used for interaction during the game, as shown in Fig.  4. The origin of this reference system corresponds to the virtual home position. A printed ArUco marker, mounted on a rigid support, is used to define the pose of this reference system^35,36^.

Before the game session, the Kinect camera acquires a single RGB-D frame and computes the pose of the ArUco reference system with respect to the Kinect camera reference frame. During the game session, the ArUco marker is removed, and the system is able to compute the position of the subject’s hand with respect to the ArUco reference system, which is then used to control the virtual hand in the virtual scene.

**Hand calibration.** Once the virtual space has been correctly positioned and oriented with respect to the subject, a second fundamental calibration step is performed. This phase aims to determine the gain that enables dimensional matching between the real and virtual worlds. In other words, since the size of the virtual world–i.e., the position of virtual objects within the environment–does not change with the subject, the 3D position of the real hand must be scaled along the three axes before being applied to the virtual hand.

This is necessary for two main reasons: (1) the Kinect libraries compute hand position in meters, whereas the Unity framework operates in a virtual space without predefined measurement units; and, more importantly, (2) it allows the dimensions of the real play space to be adapted to the specific anthropometric characteristics of the player’s upper limb.

Upon initiation, an on-screen prompt instructs the subject to extend their arm forward, simulating the action of reaching for an object. The assisting operator can easily adjust the scaling factor manually through a simple panel in the serious game. The operator determines that the correct gain has been found when the virtual hand is able to reach a specific virtual wall within the scene when the subject’s upper limb is fully extended forward.

#### Performance metrics

A set of metrics was proposed, computed, and evaluated to quantify each subject’s performance, whence the VMA capability. These indices have been extracted for each subject, trial, and mapping condition.

The main metrics consider the percentage of Collected Targets (CT) and the execution time (TIME) to quantify the duration of the trial. CT ranges between 0 and 1, where 1 indicates that 100% of the targets are taken. However, these metrics do not describe the trajectory followed by the subject during the trials.

**Fig. 5:**
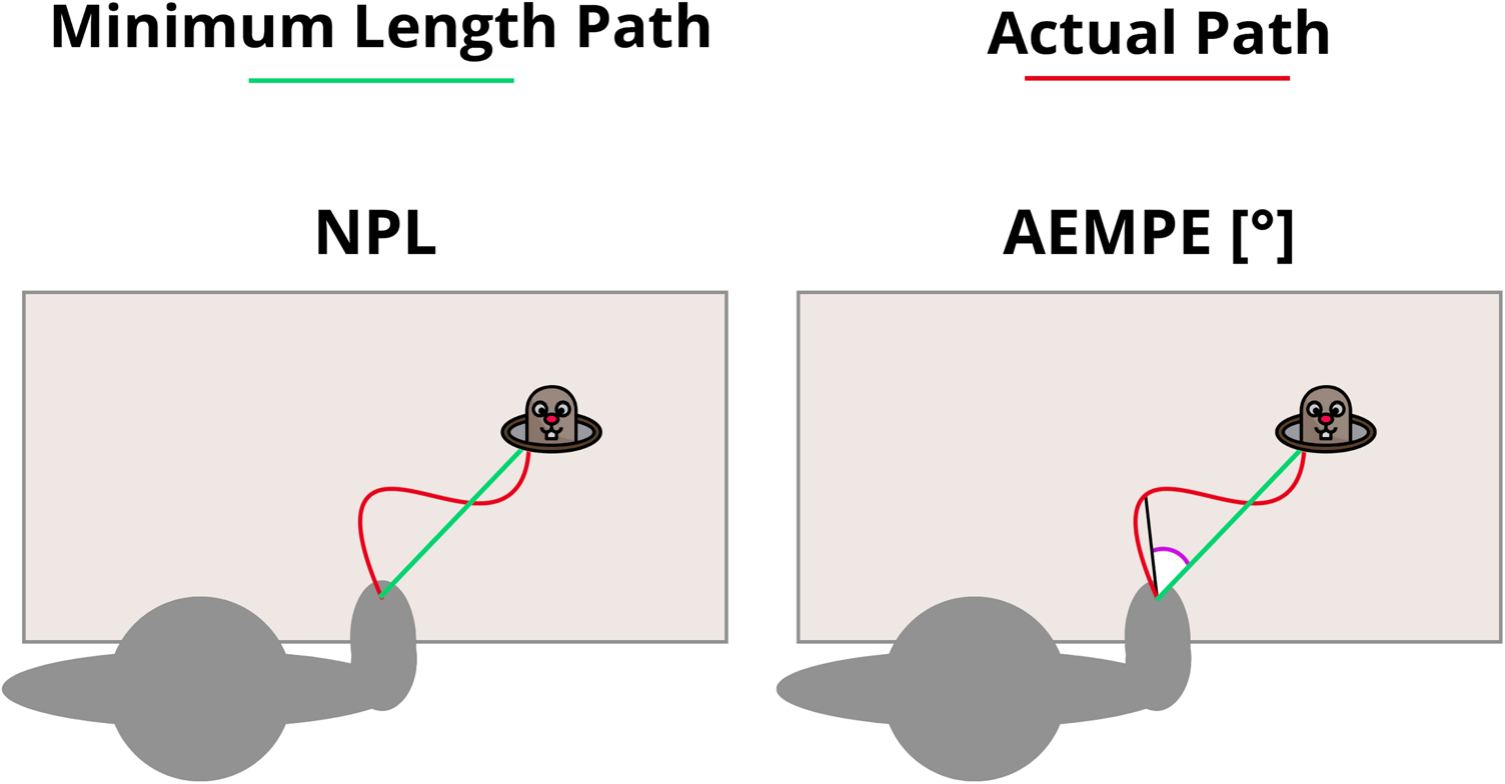
Illustration of the performance metrics used to quantify VMA during the reaching tasks in virtual reality. Left: The NPL is computed as the ratio between the actual path (red) and the MLP (green), defined as the straight line connecting the subject’s starting position and the target. Right: The AEMPE is defined as the angle (purple) between the MLP and the segment (black) connecting the subject’s initial position to the point on the actual path corresponding to the highest peak of the perpendicular error signal.

Therefore, kinematic metrics (see Fig. 5) have been extracted from the path followed by the virtual hand in the reaching tasks. The choice of computing the kinematic metrics on data generated by the virtual environment is usually adopted to avoid possible dependency with the anthropometric characteristics of the subject. The Normalized Path Length (NPL) is computed as the division of the actual path length and the length of the minimum path (MLP) connecting the initial and target positions^26^. The SPEED is defined as the division of the actual path length by TIME. The smoothness of movement is computed using the Spectral Arc Length (SPARC) method^37^. It is considered a robust and reliable indicator of motor smoothness, as it is minimally affected by noise or movement duration, and it has been validated in both healthy and clinical populations^38,39^. Infact, it quantifies the intermittency of motion by capturing fluctuations due to accelerations and decelerations. This metric is influenced by multiple factors, including: the subject’s motor control capabilities–often linked to task familiarity and environmental adaptation in healthy individuals; the presence of neurological impairments or musculoskeletal limitations, as in the case of patients with CP; and specific task characteristics, which may introduce discontinuities in the velocity profile to accommodate restricted ranges of motion. The Angle Error at Maximum Path Error (AEMPE) is the angle at which the hammer position is at the maximum distance from the MLP. This index is used to assess directional errors and changes in direction. The indexes dependent on the virtual path length, such as NPL and TIME, are expressed in Unity measurement units.

*Absolute and relative differences.*

In addition to the absolute values, for each metric, absolute differences are computed as the difference between the metric value in each perturbed mapping condition and the value in the *No Perturbations* condition, whilst relative differences are obtained by dividing the absolute differences by the corresponding value in the *No Perturbations* condition. Accordingly, both absolute and relative measures consider the following differences:

1. ’Rev. vs NP’ is the comparison between the metric value in the *Reversal* condition and the one in the *No Perturbations* condition.
2. ’Gain. vs NP’ is the comparison between the metric value in the *Gain* condition and the one in the *No Perturbations* condition.
3. ’Rev. + Gain vs NP’ is the comparison between the metric value in the *Reversal+Gain* condition and the one in the *No Perturbations* condition.

### Experiments

The proposed framework was systematically evaluated to verify both the robustness of the game implementation and the informativeness of the features in capturing task difficulty associated with mapping perturbations. Two cohorts of patients with cerebral palsy and control subjects were recruited and asked to follow a structured experimental protocol. Ultimately, the extracted features, which reflect performance across different mapping conditions, were statistically compared and analyzed among conditions and between groups.

#### Participants

The study was conducted in accordance with the ethical principles outlined in the Declaration of Helsinki and its later amendments. The research protocol, including all procedures for data collection, management, and confidentiality, was reviewed and approved by the Ethics Committee of Area Vasta Centro of Tuscany Region (approval number: CET 44/2023). Written informed consent was obtained from all participants (or from parents/legal guardians for minors or individuals unable to provide consent) prior to inclusion in the study. All data were anonymized before analysis to ensure participant privacy and confidentiality. The study involved two distinct cohorts to assess differences in VMA capabilities during upper-limb reaching tasks under controlled experimental conditions. The subjects of the Patient Group were enrolled from inpatients or outpatients belonging to the Pediatric department of IRCCS Fondazione Don Gnocchi ETS of Florence. The control subjects were recruited from an age-matched convenience sample of peers. Each participant underwent a preliminary standardized neuropsychological assessment to ensure that all included children met the study’s inclusion criteria in terms of cognitive functioning , attention , and working memory using the Italian version of the Wechsler Intelligence Scale for Children – Fourth Edition (WISC-IV)^40^. Attention was assessed through selected subtests of the Italian version of the NEPSY-II battery^41^, including Visual Attention, Auditory Attention, and Response Set. Executive and inhibitory functions were evaluated using the Inhibition subtest from the same battery. All tests employed are standardized and based on age-specific normative cut-offs.

The inclusion criteria comprised patients with spastic diplegic cerebral palsy, diagnosed clinically and confirmed by magnetic resonance imaging (MRI), as well as control participants with typical psychomotor development. Eligible participants were between 10 and 18 years of age and were required to demonstrate, as assessed by a clinician, the ability to perform with both upper limbs the movements necessary to engage with the game.

Exclusion criteria included a history of multilevel upper limb surgery and the administration of botulinum toxin injections in the upper limb within the previous six months. Participants with central visual impairments that could interfere with video game interaction were also excluded.

For each participant with CP, the following clinical characteristics were assessed: Gross Motor Function Measure (GMFM)^42^, Manual Ability Classification System (MACS)^43^, and the presence of a perceptual disorder. A perceptual disorder was identified when at least three of the following clinical signs were observed during spontaneous gait: startle reaction, upper limbs in a startle position, gaze deviation outside the visual field, frequent blinking, grimacing, and postural freezing^44^.

**Table 1:** Demographic and clinical characteristics of the Patient Group and the Control Group.

Variable	Patient group (n = 15)	Control group (n = 10)
Age (years), mean ± SD	12.1 ± 3.5	10.1 ± 1.5*
Sex (M / F)	10 / 5	6 / 4 **
GMFM, median [IQR]	3 [2–4]	—
MACS (Level I / II / III / IV)	3 / 5 / 3 / 4	—
Perceptual Disorder, n (%)	7 (46.7%)	—

* Age: p = 0.064 (independent sample t-test) ** Sex: p = 1.000 ($\chi ^2$)(no baseline difference - *α =0.05*).

Table 1 shows demographical and clinical characteristics of CP Patient Group and Control Group. The Patient Group comprised 15 individuals (mean age: *12.1 ± 2.5* years; 5 females), the Control Group consisted of 10 age and sex-matched healthy participants (mean age: *10.1 ± 1.5* years; 4 females), with no known neurological or musculoskeletal impairments.

Each participant received instructions on how to perform the task and was briefed on the game mechanics, without being informed about the presence of visual perturbations, so as to avoid any bias in their performance due to prior awareness.

#### Experimental protocol

The academic authors and the clinicians of the IRCCS Fondazione Don Carlo Gnocchi ETS arranged the experimental protocol to simulate realistic hand motion in a subject-tailored Serious Game. Therefore, the game session has been preceded by the measurement of participants’ parameters, including the subject’s upper limb and hand range of motion.

**Fig. 6:**
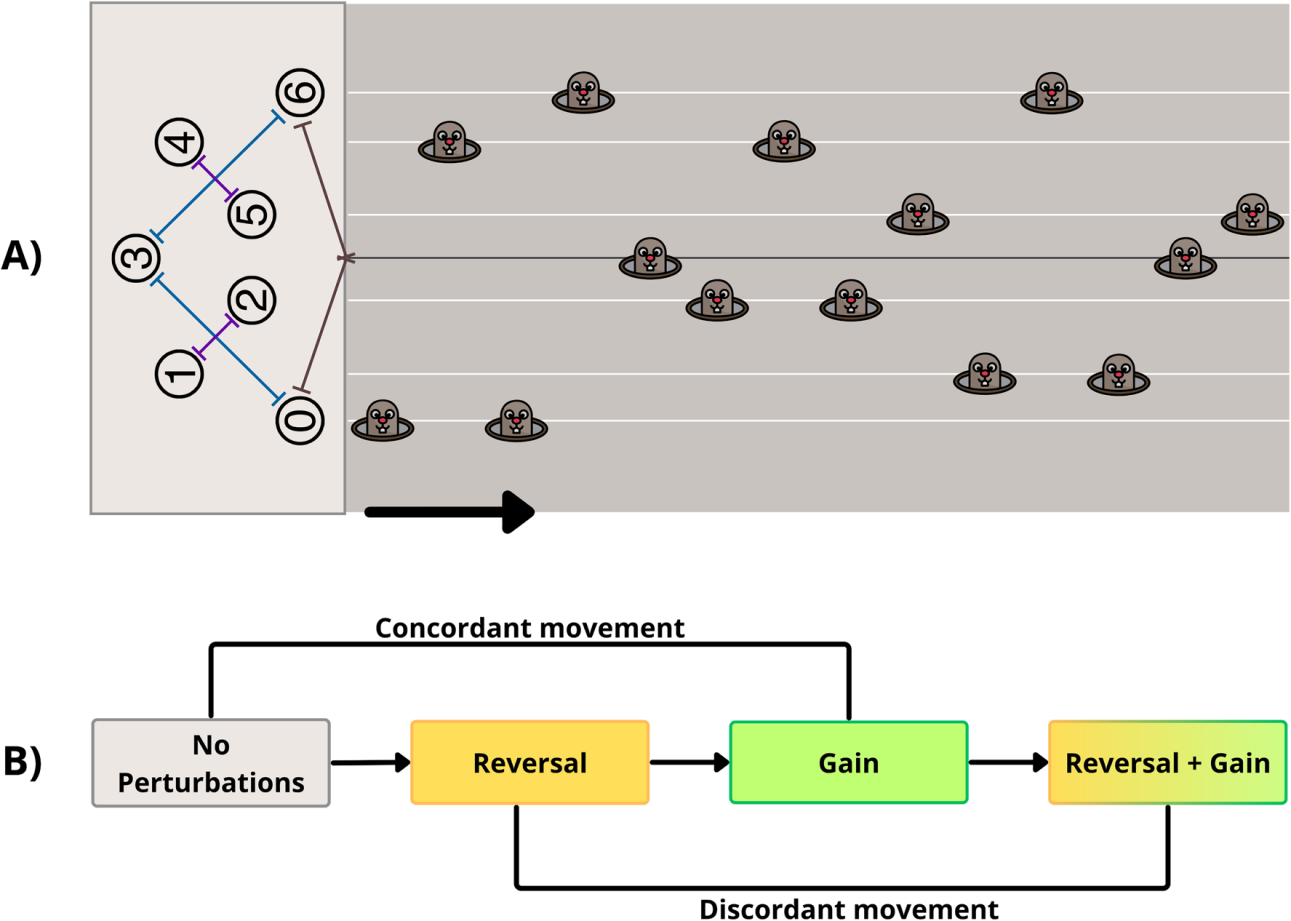
(**A**) Temporal sequence of target appearances. The sequence is fixed across the four blocks. The arrow denotes the direction of the mole spawning order. (**B**) Sequence of the four blocks defined by the experimental protocol and associated with the mapping conditions.

A customized protocol has been defined to test the workflow; notwithstanding, different protocols with different mole placement or visual perturbations can be employed based on the needs. The protocol of the presented study is made up of four blocks. Each block includes the same target sequence of fourteen reaching trials, as shown in Fig. 6A.

Each block entails a visuomotor perturbation determining a different mapping condition, thus leading to the following sequence of conditions, as shown in Figure 6B: (1) *No Perturbations*, (2) *Reversal*, (3) *Gain*, and (4) *Reversal+Gain*. In the first and third blocks, the lateral movement of the virtual hand is concordant with that of the physical hand, whereas in the second and fourth blocks a directional mismatch is introduced. Specifically, in the first block, the virtual hand replicates the physical motion on the ML axis without any amplification or inversion. The second block implements the *Reversal* condition, in which the direction of the virtual hand’s motion is inverted relative to the physical hand. In the third block, the *Gain* condition is introduced by amplifying the lateral displacement of the virtual hand with a specific gain factor, effectively reducing the required physical range of motion by approximately 50%. Finally, in the fourth block, both perturbations are combined: the virtual hand’s lateral movement is simultaneously inverted and amplified using the same gain factor as in the third block.

This fixed succession affects the outcomes due to learning effects; however, this protocol is not designed for a standard study of motor learning/control. On the other hand, this work aims to assess VMA capabilities in healthy and pathological subjects through visual perturbations and whether the effect of these latter ones is captured by the proposed performance indexes.

### Comparisons and statistical analysis

Here we describe all the conducted comparisons and statistical analysis that have been performed using MATLAB 2024b and its toolboxes.

*Correlation analysis between functional upper limb levels and performance metrics in Patient Group.*

In the Patient Group, a purely exploratory analysis (due to the small subgroups sample size) was conducted about the relationship between MACS levels and performance metrics across experimental conditions. Spearman’s rank correlation test was applied to both Absolute and Relative Differences. Correlations were considered relevant when the correlation coefficient was $\rho \ge 0.5$ and the significance level was *p < 0.05*.

*Metric absolute values.* A qualitative analysis has been performed through visual inspection of the boxplots representing each metric distribution across experimental conditions. This approach allowed the identification of general trends, variability patterns, and potential condition-related differences within each group. Given that the primary aim of this study is to investigate differences between groups rather than to provide a detailed analysis of within-group condition effects, the qualitative inspection on absolute values was considered sufficient to describe trends across conditions. Between-group comparisons of absolute values have been conducted separately for each variable and experimental condition with independent sample t-test or Mann–Whitney U test when the assumption of normality was met or not, respectively. The normal distribution of each index has been verified by applying the Shapiro-Wilk test with a significance level of α = 0.05.

*Metric absolute and relative difference values.*

Concerning the absolute and relative differences, the Friedman test for non-parametric variables has been applied for within-group comparison. Such within-group analysis has not been further explored with post-hoc tests, as the primary aim of the study is the comparison between groups rather than an in-depth understanding of differences between conditions within each group. The Friedman test has been performed only to verify whether the considered metrics are able to capture the varying difficulty introduced by the visuomotor perturbations.

Between-group analysis was conducted separately for each variable and experimental condition. Depending on whether the normality assumption was satisfied, either a t-test or a Mann–Whitney U test was applied. Normality for each index was assessed using the Shapiro–Wilk test with a significance level of α = 0.05.

## Results

### Correlation between MACS levels and performance metrics

Higher MACS levels, reflecting greater upper limb impairment, were significantly associated with larger changes in SPEED values across conditions, in both Absolute (Reversal vs No Perturbation, *ρ =0.66*, *p = 0.007* - Gain+Reversal vs No Perturbation, *ρ = 0.52*, *p = 0.048*) and Relative Differences (Reversal vs No Perturbation, *ρ =0.68*, *p = 0.006*, Gain vs No Perturbation, *ρ = 0.54*, *p = 0.034* - Gain+Reversal vs No Perturbation, *ρ = 0.54*, *p = 0.040*). This suggests that more impaired patients could rely on compensatory strategies, particularly modulation of movement speed, to accomplish the task. No significant correlations were observed for other metrics.

### Comparisons concerning the metric absolute values

**Fig. 7:**
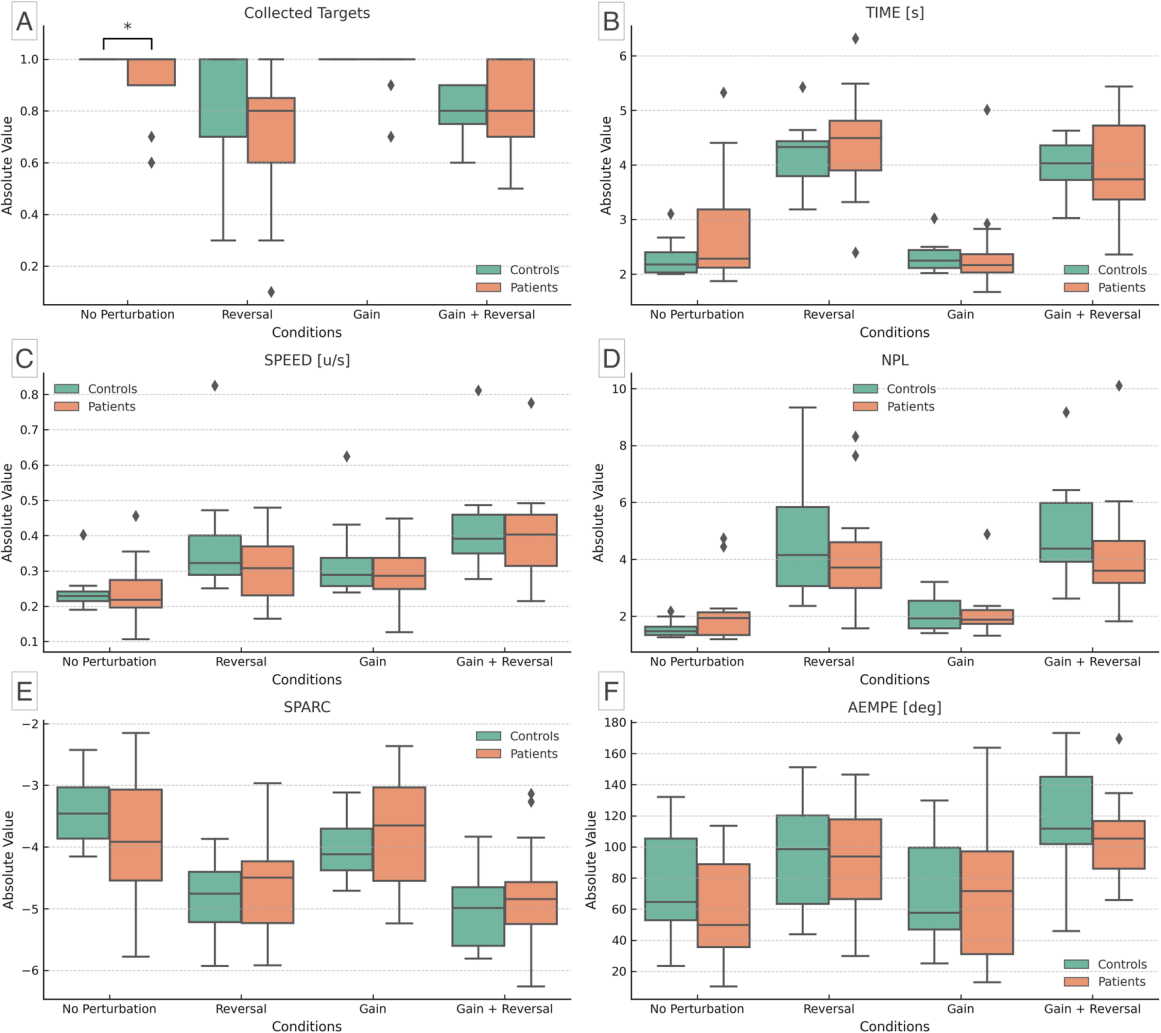
Between-group comparisons of the absolute values for each metric were performed across different conditions. An independent samples t-test or a Mann–Whitney U test was used, when the assumption of normality was met or not, respectively. * Represents statistical significance set at *p<0.05*. (**A**): Collected targets; (**B**): TIME; (**C**): SPEED; (**D**): NPL; (**E**): SPARC; (**F**): AEMPE.

Regarding the comparison across conditions within each group, a qualitative analysis was conducted. Figure 7 shows, for each metric and each group, the boxplots of the absolute metric values (Collected Targets, TIME, SPEED, NPL, SPARC, and AEMPE) recorded under each gaming condition (No Perturbation, Reversal, Gain, Reversal+Gain). The inspection of this figure indicates that the different conditions introduce varying levels of difficulty, thereby supporting the validity of the implemented visuomotor perturbations.

Overall, it can be observed that conditions involving inversion, i.e., Reversal and Reversal+Gain, lead to worse metric values compared to conditions without inversion, i.e., No Perturbation and Gain. This trend is consistent across both the Control Group and the Patient Group. This difference is more pronounced for some metrics (e.g., CT, TIME, and NPL) and less marked for others (e.g., SPEED, SPARC, and AEMPE). When comparing the Gain condition with the No Perturbation condition, it emerges that Gain leads to slightly worse performance only for a few metrics, specifically SPEED and NPL.

With regard towhilst the controls achieved the same result the quantitative comparison between the two groups for each condition, no substantial differences emerged from the overall analysis. The most notable differences between the Control Group and the Patient Group were observed in the No Perturbation condition, specifically for the metrics CT, TIME, and SPARC. These results indicate that control participants were generally able to reach the targets more effectively and efficiently in that condition. Statistical analysis revealed no significant differences overall, with the exception of the CT metric in the No Perturbation condition, which reached statistically significant (*p = 0.022*).

### Comparisons concerning the metric absolute and relative difference values

**Fig. 8:**
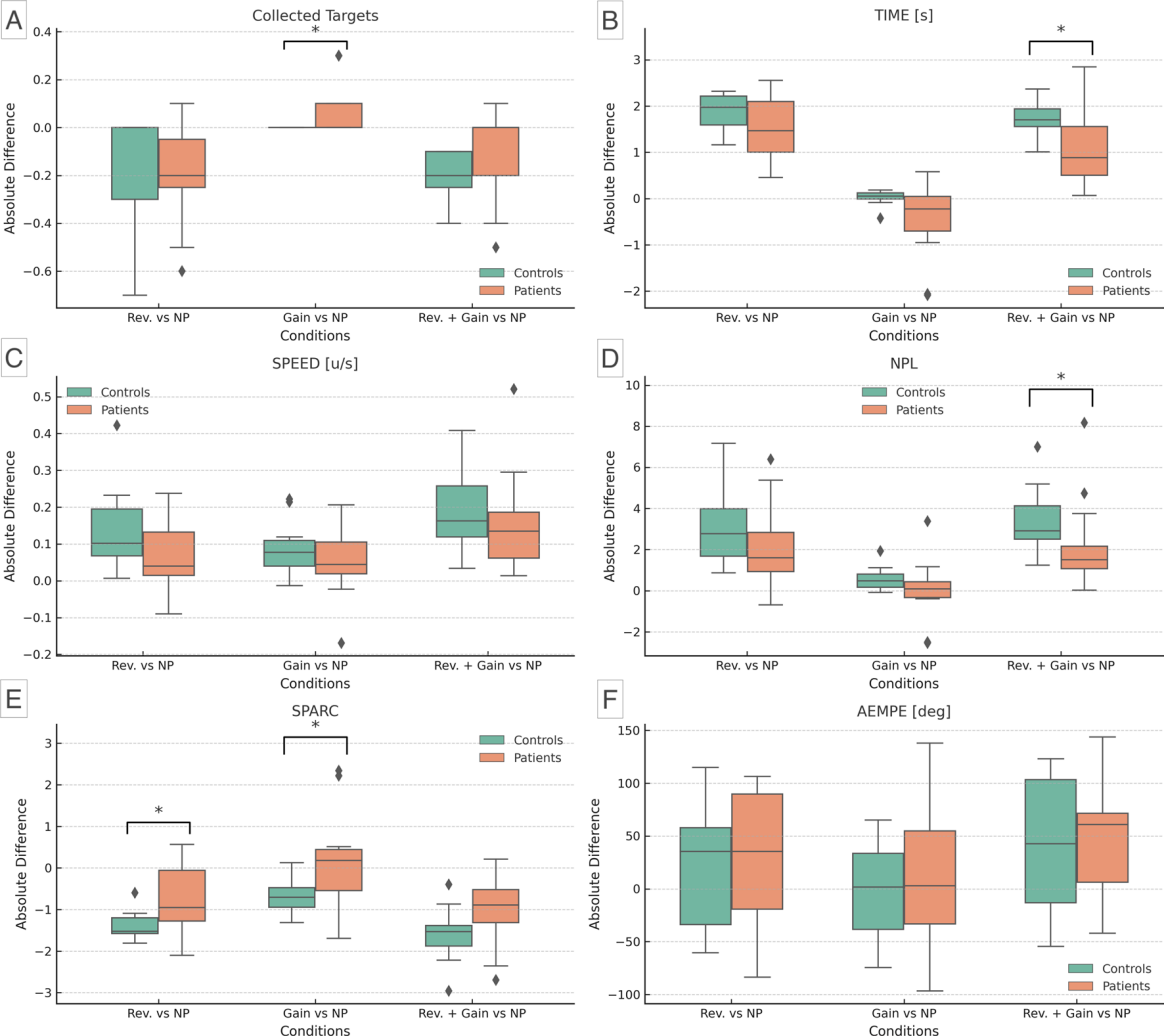
Between-group comparisons of the Absolute Difference Values of each metric in different conditions, with * representing statistically significant comparisons with *p<0.05* with respect to No Perturbation. (**A**): Collected targets; (**B**): TIME; (**C**): SPEED; (**D**): NPL; (**E**): SPARC; (**F**): AEMPE.

**Fig. 9:**
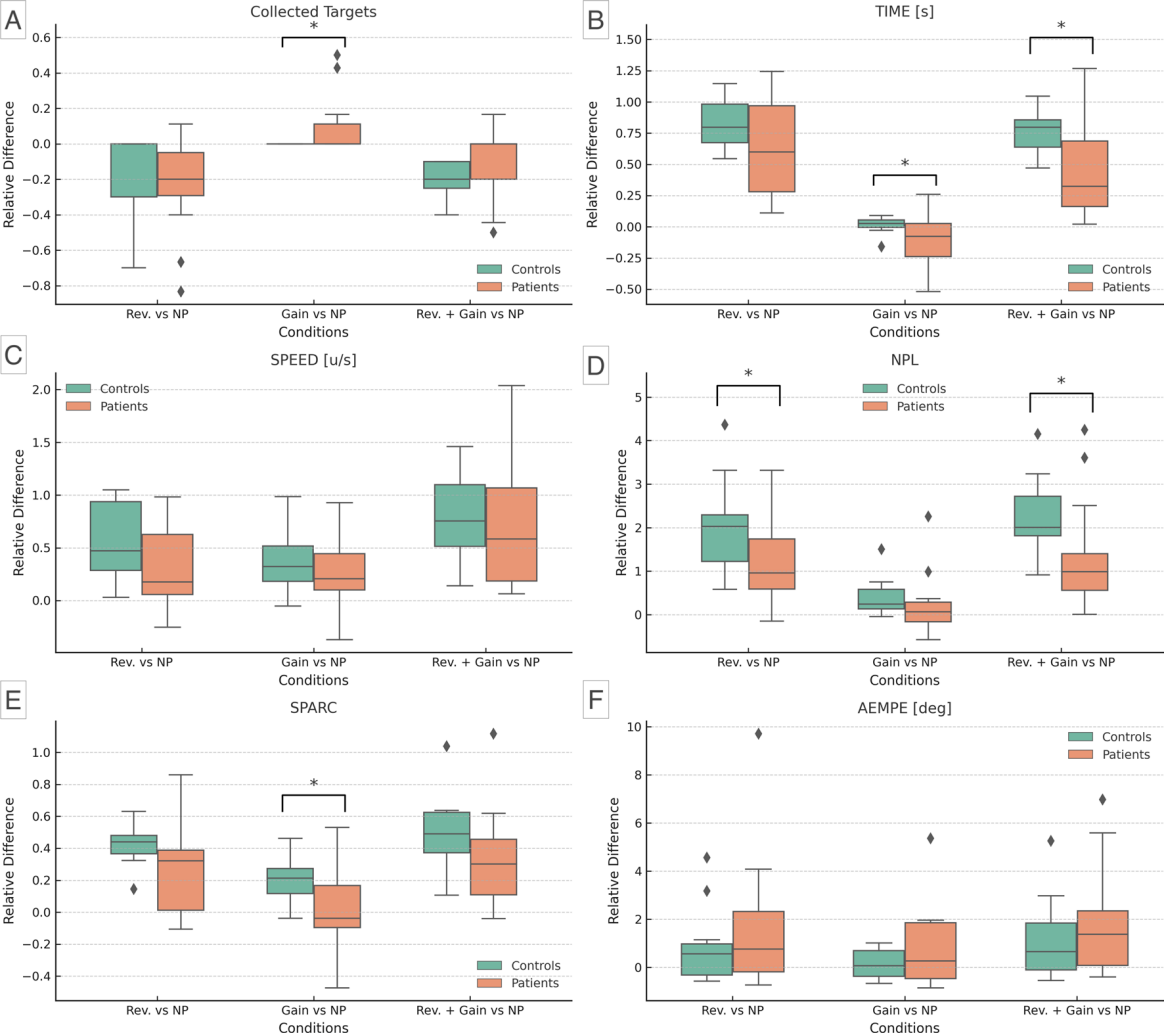
Between-group comparisons of the Relative Difference Values of each metric in different conditions, with * representing statistically significant comparisons with *p<0.05* with respect to No Perturbation. (**A**): Collected targets; (**B**): TIME; (**C**): SPEED; (**D**): NPL; (**E**): SPARC; (**F**): AEMPE.

This section presents the the comparisons of the absolute and relative differences in the metrics considering mapping conditions in pairs. Three main differences were considered: Reversal vs No Perturbation, Gain vs No Perturbation, and Reversal+Gain vs No Perturbation. Figures 8 and 9 show the boxplot distribution for each metric for the absolute and relative differences, respectively. Table 2 and Table 3 report, for all considered differences, the median, Q1 and Q3 values, and the p-value of the within-group test for the Control Group and the Patient Group, respectively.

**Table 2:** Controls.

Variable	Rev. vs NP	Gain vs NP	Rev. + Gain vs NP	*p*-value
Absolute differences				
Collected targets	$-0.30\ [-0.30,\ 0.00]$	$0.00\ [0.00,\ 0.00]$	$-0.20\ [-0.25,\ -0.10]$	0.002
TIME [s]	$1.97\ [1.59,\ 2.21]$	$0.05\ [-0.01,\ 0.12]$	$1.71\ [1.56,\ 1.94]$	< 0.001
SPEED [u/s]	$0.10\ [0.07,\ 0.20]$	$0.08\ [0.04,\ 0.11]$	$0.16\ [0.12,\ 0.26]$	0.038
NPL	$2.77\ [1.68,\ 3.98]$	$0.48\ [0.17,\ 0.80]$	$2.92\ [2.51,\ 4.14]$	0.001
SPARC	$-1.52\ [-1.58,\ -1.20]$	$-0.71\ [-0.95,\ -0.46]$	$-1.53\ [-1.88,\ -1.38]$	0.001
AEMPE [deg]	$35.30\ [-33.76,\ 57.95]$	$1.77\ [-38.23,\ 33.62]$	$42.62\ [-13.08,\ 103.43]$	0.020
Relative differences				
Collected targets	$-0.30\ [-0.30,\ 0.00]$	$0.00\ [0.00,\ 0.00]$	$-0.20\ [-0.25,\ -0.10]$	0.002
TIME [s]	$0.82\ [0.67,\ 0.92]$	$0.01\ [-0.01,\ 0.04]$	$0.76\ [0.65,\ 0.89]$	< 0.001
SPEED [u/s]	$0.55\ [0.36,\ 0.68]$	$0.3\ [0.18,\ 0.43]$	$0.77\ [0.53,\ 1.01]$	0.038
NPL	$2.03\ [1.26,\ 2.80]$	$0.39\ [0.11,\ 0.60]$	$2.27\ [1.76,\ 3.40]$	0.001
SPARC	$0.42\ [0.35,\ 0.52]$	$0.20\ [0.13,\ 0.31]$	$0.50\ [0.39,\ 0.62]$	0.001
AEMPE [deg]	$0.84\ [-0.25,\ 1.88]$	$0.12\ [-0.75,\ 0.84]$	$1.19\ [0.03,\ 2.64]$	0.020

Within-group comparisons of absolute and relative differences across experimental conditions. Values are reported as median [Q1, Q3]. Statistical significance was assessed using the Friedman test.

**Table 3:** Patients.

Variable	Rev. vs NP	Gain vs NP	Rev. + Gain vs NP	*p*-value
Absolute differences				
Collected targets	$-0.20\ [-0.25,\ -0.05]$	$0.00\ [0.00,\ 0.10]$	$0.00\ [-0.20,\ 0.00]$	< 0.001
TIME [s]	$1.46\ [1.00,\ 2.10]$	$-0.23\ [-0.70,\ 0.05]$	$0.89\ [0.50,\ 1.56]$	< 0.001
SPEED [u/s]	$0.04\ [0.01,\ 0.13]$	$0.04\ [0.02,\ 0.10]$	$0.14\ [0.06,\ 0.19]$	0.002
NPL	$1.61\ [0.93,\ 2.84]$	$0.08\ [-0.34,\ 0.42]$	$1.51\ [1.07,\ 2.165]$	< 0.001
SPARC	$-0.95\ [-1.28,\ -0.06]$	$0.18\ [-0.54,\ 0.44]$	$-0.89\ [-1.32,\ -0.53]$	< 0.001
AEMPE [deg]	$47.26\ [-22.25,\ 91.01]$	$9.28\ [-41.36,\ 30.91]$	$45.85\ [-14.27,\ 118.50]$	0.004
Relative differences				
Collected targets	$-0.20\ [-0.29,\ -0.05]$	$0.00\ [0.00,\ 0.11]$	$0.00\ [-0.20,\ 0.00]$	< 0.001
TIME [s]	$0.70\ [0.50,\ 0.87]$	$-0.15\ [-0.39,\ 0.02]$	$0.54\ [0.33,\ 0.85]$	< 0.001
SPEED [u/s]	$0.24\ [0.13,\ 0.50]$	$0.25\ [0.10,\ 0.35]$	$0.54\ [0.29,\ 0.71]$	0.002
NPL	$0.99\ [0.51,\ 2.12]$	$0.07\ [-0.34,\ 0.29]$	$1.12\ [0.68,\ 1.66]$	< 0.001
SPARC	$0.28\ [0.09,\ 0.43]$	$0.01\ [-0.25,\ 0.21]$	$0.27\ [0.15,\ 0.45]$	< 0.001
AEMPE [deg]	$0.89\ [-0.20,\ 2.43]$	$0.09\ [-0.83,\ 0.61]$	$0.97\ [-0.12,\ 2.74]$	0.004

Within-group comparisons of absolute and relative differences across experimental conditions. Values are reported as median [Q1, Q3]. Statistical significance was assessed using the Friedman test.

#### Within-group analysis

The analysis of the within-group variability (Tables 2 and 3) confirms that game conditions systematically influence the sensorimotor performance. In both groups, all variables were significantly modulated among mapping conditions, which reflects the sensitivity of the task to contextual changes. In the Control Group, although all variables showed significant changes, the magnitude of variation was generally smaller. The CT metric was equal to 100% in No Perturbation and Gain condition for all control subjects. In the Patient Group, significant differences were also observed among all variables. The most prominent effects were found for NPL and AEMPE, with consistent significance in both absolute and relative measures. Note that patients performed better in terms of CT and TIME in the Gain than in the No Perturbation condition. Both groups exhibited the worst performance in terms of CT within the conditions that included a Reversal perturbation (i.e., Rev and Rev+Gain). The Reversal condition increased the TIME required to reach the targets, but also elicited kinematic adjustments in terms of increased trajectory length, (i.e., NPL), and movement directional error (i.e., AEMPE).

#### Between-group analysis

Figures 8A and 9A show the between-group Absolute and Relative Differences in Collected Targets, with respect to the mapping conditions. A significant difference was only found between No Perturbation condition vs Gain condition for both Absolute and Relative differences (*p = 0.022* in both cases), as the patients’ performance was slightly better in the Gain condition respect to No Perturbation, whilst the controls achieved the same result.

Regarding TIME metric, the Absolute and Relative differences for the Patient Group are lower between the No Perturbation and Reversal+Gain conditions reveling a significant difference respect to Control Group (*p = 0.017* and *p =0.019*, respectively), whereas a trend significance are exhibited between the No Perturbation and Gain conditions (*p = 0.069* and *p = 0.004*, respectively).

Regarding the SPEED metric, no inter-group differences were found among conditions, as it can be noticed in Fig. 8C (SPEED - Absolute Difference) and Fig.  9C (SPEED - Relative Difference) (*p>0.05* Mann-Whitney U or independent t-test, as appropriate).

As shown in Fig. 8D (NPL - Absolute Difference) and Fig. 9D (NPL - Relative Difference) reporting the Absolute and Relative Differences, the NPL differences are higher for the controls than for the patients. This is particularly evident in the Reversal+Gain condition, as highlighted by the observed significant absolute differences (controls = *3.52 ± 1.59* patients = *2.1 ± 2.08*, *p=0.009*). Remarkably, compared to the No Perturbation condition, the Reversal condition leads to implement a motor strategy that increases the trajectory length by approximately tripling and doubling it for the controls and patients, respectively (Fig.  9D - NPL Relative Difference).

The SPARC index exhibits a lower relative alteration in the Patient Group than in the controls among the different game conditions, however only in Gain condition the difference is statistical significance (*p=0.039*) (see Fig.  9E - SPARC Relative Difference); this witnesses a significantly higher deterioration in movement fluidity for the pathological subjects in the conditions with Reversal (*p=0.023*) or Gain (*p=0.029*) perturbations in absolute terms (see Fig.  8E - SPARC Absolute Difference).

No significant differences (*p>0.05* Mann-Whitney U or independent t-test, as appropriate) can be noticed between Control and Patient Groups when comparing No Perturbation with the other mapping conditions in terms of absolute and relative differences of the AEMPE (see Fig.  8F—AEMPE Absolute Difference and Fig.  9F—AEMPE Relative Difference).

## Discussion

In the present study, a novel assessment framework leveraging a VR-based serious game was employed to quantitatively evaluate visuomotor adaptation performance in response to task-specific perturbations during reaching tasks. This framework integrates rapid and efficient calibration procedures, an intuitive and user-friendly software interface, and the implementation of safety measures, thereby ensuring participants’ physical and psychological well-being throughout the task.

Firstly, the experimental paradigm effectively elicited visuomotor adaptation processes, as evidenced by significant changes in the computed quantitative metrics across different perturbation conditions. The within-group analysis revealed that performance was significantly worse in conditions involving movement inversion, in both experimental groups. In contrast, the Gain condition produced a significant facilitation effect in the CP group. These findings suggest that the proposed system is sensitive to modifications in sensorimotor behavior (i.e., reaching strategy mechanisms) in both healthy and clinical populations.

The between-group analysis of absolute variable values related to movement perturbation effects showed that the Control Group exhibited better performance in reaching the target in the No Perturbation condition. On the other hand, the between-group analysis of absolute and relative differences in the variable values suggests the following observations.

The Gain condition induces a substantial variation in the CP group compared to the Control Group, acting as a facilitating factor in contrast to the Reversal condition. Specifically, movement amplification in the Gain condition appears to facilitate target achievement in the CP group. This effect may be interpreted as the Gain condition enabling CP participants to reach the target with a reduced range of motion. This interpretation is supported by the results of the NPL metric. Healthy individuals tend to use more variable (i.e., less repeatable) trajectories to reach targets during upper-limb reaching tasks, as found in this study under the No Perturbation condition and reported in previous studies^45^. In the Reversal condition, healthy participants exhibit longer trajectories (i.e., higher NPL) as a strategy to reach the target. This may suggest the adoption of a broader motor exploration strategy, enabled by their higher level of motor control, to identify the most efficient movement direction.

This hypothesis is further supported by the analysis of movement smoothness, assessed through the SPARC metric, which shows relative differences between the No Perturbation and Gain conditions in the healthy group. The SPARC value is influenced by multiple factors, including motor control capabilities, which are typically enhanced by task familiarity and adaptive responses to environmental demands in healthy individuals. In clinical populations, particularly in individuals with neurological impairments such as cerebral palsy, alterations in SPARC may instead reflect discontinuities in the velocity profile associated with compensatory mechanisms, including those related to spasticity or abnormal co-contractions^37^. Indeed, the patients’ outcomes in terms of both SPARC and NPL suggest a “movement reduction” strategy, likely aimed at facilitating motor control under visuomotor perturbations. Such a strategy is reminiscent of the phenomenon of “freezing,” characterized by reduced movement amplitude and simplified motor patterns to cope with impaired neuromuscular and/or perceptual control, as commonly reported in individuals with CP^46,47^.

Concerning the TIME metric, between-group absolute differences were statistically significant only in the combined Reversal+Gain condition, where the Control Group showed a larger increase in time. A plausible explanation is that the smaller increase observed in the CP group is due to their higher TIME values already recorded in the No Perturbation condition. The analysis of relative differences of the TIME variable confirms that the Gain condition facilitates target achievement in the CP group. In this group, the prolonged movement duration and the aforementioned metrics observed in the No Perturbation condition likely reflect a strategy aimed at increasing control. These findings underline how children with CP require more time to perform upper-limb movements compared to typically developing peers^48,49^.

Regarding average movement speed, i.e., SPEED, this metric remained statistically consistent across the two groups, thus limiting the understanding of the specific adaptation strategy employed. This finding aligns with related works indicating that the average velocity is not always a sensitive indicator of motor adaptation or performance quality in visuomotor tasks^50^. However, a correlation between absolute and relative SPEED value differences and MACS levels has been found suggesting that higher functional impairment leads to larger changes in speed when dealing visuomotor perturbations.

The analysis of the AEMPE metric indicates that, in the Control Group, perturbations led to changes in the initial movement direction. This suggests that healthy participants may adopt a strategy that disregards the initial direction and instead relies on multiple directional adjustments (as reflected in SPARC) and longer trajectories (higher NPL) to reach the target. In the CP group, perturbations also affected the initial movement direction, particularly in the Reversal condition, leading to wider movement trajectories compared to baseline. Given that SPARC and NPL exhibited smaller changes in CP participants than in controls, the CP group appears to adopt a strategy characterized by smaller movements and minor directional adjustments.

The proposed investigation could be further strengthened by a future recruitment campaign aimed at increasing the sample size and refining the experimental protocol. In particular, adopting a non-fixed sequence of mapping conditions–such as those commonly used in motor control and learning studies–could help reduce biases due to learning effects. However, a fixed sequence has been adopted in the present study to ensure consistency across participants and enable a more reliable comparison among groups, which represents the primary aim of the work. Although this design may introduce residual effects, the observed metric absolute value trends across blocks suggest that performance variations are mainly driven by the imposed visuomotor perturbations rather than by practice-related effects over time.

## Conclusions

This study shows that two groups of subjects–one with Typical Development and one with Cerebral Palsy (CP)–adopt opposite strategies in response to different visuomotor perturbations that consisted in reverting and/or amplifying virtual displacements during a reaching task. The Control Group uses longer trajectories and frequent direction changes, whereas the CP group employs a more conservative, reduced-movement strategy to explore space and facilitate motor control.

Despite these divergent strategies, both groups achieved similar levels of performance in the task. Since the upper limb involved in CP participants was the less impaired one, these findings may reflect differences in motor planning strategies during a reaching task, with minimal confounding effect from motor impairment severity. Indeed, in the CP group, motor control based on feedback mechanisms may be impaired or partially effective. Therefore, reduction of movement could be a strategy to optimize visual and sensory information that underlies the feedback control. Future studies should investigate which metrics or game conditions can better capture difficulties in motor planning or in the perception of movement awareness.

## Data Availability

The data presented in this study are available on request from the corresponding author.
